# The molecular basis of beta-thalassemia intermedia in southern China: genotypic heterogeneity and phenotypic diversity

**DOI:** 10.1186/1471-2350-11-31

**Published:** 2010-02-25

**Authors:** Wanqun Chen, Xinhua Zhang, Xuan Shang, Ren Cai, Liyan Li, Tianhong Zhou, Manna Sun, Fu Xiong, Xiangmin Xu

**Affiliations:** 1 Department of Medical Genetics, School of Basic Medical Sciences, Southern Medical University, Guangzhou, Guangdong 510515, China; 2 Department of Hematology, 303 Hospital of People's Liberation Army of China, Nanning, Guangxi 530021, China; 3 Department of Medical Genetics, Liuzhou Municipal Maternity and Child Healthcare Hospital, Liuzhou, Guangxi 545001, China; 4 Department of Biochemistry, Medical College, Jinan University, Guangzhou, Guangdong 510632, China

## Abstract

**Background:**

The clinical syndrome of thalassemia intermedia (TI) results from the β-globin genotypes in combination with factors to produce fetal haemoglobin (HbF) and/or co-inheritance of α-thalassemia. However, very little is currently known of the molecular basis of Chinese TI patients.

**Methods:**

We systematically analyzed and characterized β-globin genotypes, α-thalassemia determinants, and known primary genetic modifiers linked to the production of HbF and the aggravation of α/β imbalance in 117 Chinese TI patients. Genotype-phenotype correlations were analyzed based on retrospective clinical observations.

**Results:**

A total of 117 TI patients were divided into two major groups, namely heterozygous β-thalassemia (n = 20) in which 14 were characterized as having a mild TI with the Hb levels of 68-95 g/L except for five co-inherited ααα^anti-3.7 ^triplication and one carried a dominant mutation; and β-thalassemia homozygotes or compound heterozygotes for β-thalassemia and other β-globin defects in which the β^+^-thalassemia mutation was the most common (49/97), hemoglobin E (HbE) variants was second (27/97), and deletional hereditary persistence of fetal hemoglobin (HPFH) or δβ-thalassemia was third (11/97). Two novel mutations, Term CD+32(A→C) and Cap+39(C→T), have been detected.

**Conclusions:**

Chinese TI patients showed considerable heterogeneity, both phenotypically and genotypically. The clinical outcomes of our TI patients were mostly explained by the genotypes linked to the β- and α-globin gene cluster. However, for a group of 14 patients (13 β^0^/β^N ^and 1 β^+^/β^N^) with known heterozygous mutations of β-thalassemia and three with homozygous β-thalassemia (β^0^/β^0^), the existence of other causative genetic determinants is remaining to be molecularly defined.

## Background

β-thalassemia is one of the most common monogenic disorders in the world. The incidence for this disease is high in tropical and subtropical areas including southern China. In southern China, the carrier rate of β-thalassemia is 2.54% in Guangdong [1] and 6.78% in Guangxi [2] where two provinces were thalassemia occurred most frequently. The hemoglobin E (HbE) is one of the common β-thalassemia variants with carrier rates of 0.09-0.13% [1,3], and 0.19-0.21% [[2], unpuplished report from our laboratory] , respectively, in these two regions. According to the clinical phenotypes, β-thalassemia can be divided into three main types: thalassemia major (TM), thalassemia trait (TT) and thalassemia intermedia (TI). TM is a severe form that requires transfusions from infancy for survival, whereas TT is usually asymptomatic. TI is used to indicate a clinical condition of intermediate gravity between TT and TM, which encompasses a wide phenotypic spectrum spanning from mild anemia to more severe anemia and these patients require only occasional blood transfusions, if any [4,5].

Corresponding to the phenotypic diversity, the molecular basis of TI is also variable. The major genetic modifiers of β-thalassemia are genotypes of β- and α-globin and expression of γ-globin [6,7]. Some genotypic factors have been reported to affect synthesis of the γ-globin chain, such as the 3'HS1 (+179 C→T) polymorphism [8], the (AT)xNy(AT)z motif in the 5'HS2 site [9], and the (AT)x(T)y motif in the -540 region of the β-globin gene[10]. Variation of rs11886868 (T→C) in the *BCL11A *gene has also been shown to correlate with increased HbF in European TI patients [11]. In addition, those factors that can moderate globin imbalances indirectly or cause the β-thalassemia-like phenotype, such as GATA-1 [12], alpha hemoglobin stabilizing protein (AHSP) [13,14], and heme-regulated initiation factor 2 alpha kinase (HRI) [15], are also thought to contribute to the phenotypic diversity of TI. Researchers have described molecular characterization of TI in Iranian [16], Indian [17], Italian [4,18], Israelis [19] and other populations [20]. However, to date, the genetic basis of TI in Chinese patients is still poorly understood [21]. In this study, a comprehensive analysis of the molecular basis underlying TI was performed in southern China. Genotypes of β-globin and other known modifiers linked to α/β imbalance were investigated in 117 patients with β-thalassemia intermedia phenotypes. Their clinical, hematological, and molecular data were analyzed systematically with the aim of creating a genotype-phenotype correlation. During the analysis, two novel mutations, Term CD+32(A→C) and Cap+39(C→T), were found. These two mutations were named according to the standard nomenclature rules of human hemoglobin mutation http://globin.cse.psu.edu/. Our findings provide genetic insights of TI occurrence in southern China and are useful in genetic counselling, treatment and management.

## Methods

### Subjects

A total of 117 patients from 109 families with TI phenotypes were recruited for this study and consisted of the following: 63 males and 54 females, 73 children and 44 adults. 78 patients (66.7%) came from Guangxi, 37 (31.6%) came from Guangdong, and 2 (1.7%) came from either Guizhou or Zhejiang. Their ethnic backgrounds were as follows: 71 Han, 45 Zhuang and 1 Miao. We recruited the TI patients in this study according to previously described criteria [4,5,20], in whom the classical clinical diagnosis of TI patients, such as the steady state Hb level of 60-105 g/L, age at diagnosis over two years old, and transfusion independence were emphasized for all our patients. The protocol for this study was approved by medical ethics committee of Southern Medical University. Informed consent was obtained from each individual or parents of individuals younger than 18 years old.

### Hematological analysis and Clinical data collection

Hematological data: complete blood counts and red cell indices were determined by automated cell counting (Model Sysmex F-820; Sysmex Co Ltd, Kobe, Japan); the levels of HbA, A_2 _and F were analyzed on the Bio-Rad Variant II HPLC system (HPLC, VARIAN™, Bio-Rad, Hercules, CA, USA).

Clinical data: the information about blood transfusions, thalassemia appearance, and age at diagnosis, hepatosplenomegaly and splenectomy was obtained by retrospective clinical data.

### Molecular analysis

DNA analysis: genomic DNA was extracted from peripheral blood by standard phenol/chloroform methods. The 11 known β-thalassemia mutations including -29(A→G), -28 (A→G), CD17 (A→T), β^E ^(CD26 G→A), IVS-1-1(G→T), IVS-1-5(G→C), CD27-28(+T), CD41-42 (-CTTT), CD43 (G→T), CD71-72(+A) and IVS-2- 654(C→T) [1], the two common deletions including Chinese ^G^γ^+^(^A^γδβ^0^) thalassemia [22] and Southeast Asian hereditary persistence of fetal hemoglobin (SEA-HPFH) [23], the three common α-thalassemia deletions (--^SEA^, -α^3.7 ^and -α^4.2^) [1], the six non-deletional mutations (α^cd30^,α^cd31 ^,α^cd59^,α^QS^,α^CS^, and α^WS^) [1], the ααα^anti-3.7 ^or ααα^anti-4.2 ^triplication [24,25] and *XmnI *site -158 of the ^G^γ-globin gene [26,27] were analyzed by previously described methods. Further sequence analysis was applied on both β^0^/β^0^and β^+^/β^N ^or β^0^/β^N ^samples, analyzed targets for the former (β^0^/β^0^) include both 3'HS1 and 5'HS2 core region, the promoters of the ^G^γ- and ^A^γ-globin genes, the (AT)x(T)y sequence variations at the position -540 of the β-globin gene, a single-nucleotide polymorphism (SNP) of rs11886868 in the *BCL11A *gene, as well as the whole α2- and α1-globin genes; and those for the latter (β^+^/β^N ^or β^0^/β^N^) include the core regions of both 5'HS2 and 5'HS3, the whole β-globin gene and *AHSP *gene, and the full-lenghth cDNA of GATA-1 generated by RT-PCR from mRNA. Since HRI has been shown to modify the phenotypic severity of β-thalassemia in murine models, we also sequenced the HRI cDNA in β^+^/β^N ^or β^0^/β^N ^samples by RT-PCR. The PCR primers used in this study were previously published [1,8,9,28] or designed by us as listed in table 1. Each of the above polymorphic loci with repeat motifs in heterozygous individuals was verified by sequencing cloned PCR products.

**Table 1:** Primer sequences and their location

Primer	Sequence(5'→3')	GenBank No.	Location Nucleotide(nt)	Product length
β-540	FP: tttcccaaaacctaataagtaac RP: aacttcatccacgttcacc	NG_000007 NG_000007	nt69848-nt69870 nt70647-nt70665	818 bp
Gγ-promoter	FP:tgaaactgttgctttatagga t RP: gagcttattgataacctcagacg	NG_000007 NG_000007	nt42215-nt42236 nt42850-nt42872	657 bp
Aγ-promoter	FP:ctgctaactgaagagactaagatt RP: caaatcctgagaagcgacct	NG_000007 NG_000007	nt47259-nt47283 nt47962-nt47981	723 bp
α2-globin gene	FP:tggagggtggagacgtcctg RP: ccattgttggcacattccgg	NG_000006 NG_000006	nt33537-nt33556 nt34062-nt34621	1085 bp
α1-globin gene	FP:tggagggtggagacgtcctg RP:tccatcccctcctcccgcccctgccttttc	NG_000006 NG_000006	nt37341-37360 nt38492-38521	1180 bp
*BCL11A*	FP: tgaggagacccaaacagttaaag RP: aacccacatggcaaccaatag	NT_022184 NT_022184	nt49880-nt49902 nt50359-nt50379	500 bp
*AHSP*	FP:tgtcatgtaatagggctcagtaa RP:tggtcactcaaggctgctaac	NW_001838236.1 NW_001838236.1	nt1217371-nt1217395 nt1218685-nt1218705	1335 bp
*HRI*	FP: accccgaatatgacgaatc RP: aaggcttactaaatacaacg	NM_014413 NM_014413	nt166-nt184 nt2034-nt2053	1888 bp
*GATA-1*	FP: tgggatcacactgagcttgc RP: gctacaagaggagaaggacacc	NM_002049 NM_002049	nt10-nt29 nt1365-nt1387	1378 bp

**Table 2:** The molecular, hematological and clinical data of 117 thalassemia intermedia (TI) patients

	Molecular data				Hematological data (Mean ± SD)					Clinical data		
	**β-globin genotype**	**α-globin genotype**	**No.**	***XmnI*^*1*^**	**Hb (g/L)**	**MCV (fL)**	**MCH (pg)**	**HbA2 (%)**	**HbF (%)**	**Transfusion T:NT:NA^2^**	**Splenectomy S:NS:NH:NA^3^**	**Age at diagnosis^4^**
**I**	β^+^/β^+^	Normal	9	0:0:9:0	78.8 ± 10.6	65.0 ± 4.4	21.2 ± 1.7	4.7 ± 1.5	58.2 ± 9.8	2:5:2	2:5:0:2	2.8 ± 0.8(7)
	β^+^/β^0^	Normal	32	1:4:26:1	74.4 ± 10.0	70.0 ± 9.8	20.9 ± 2.5	4.2 ± 1.5	53.8 ± 26.9	20:10:2	9:16:5:2	5.1 ± 5.4(30)
	β^0^/β^0^	Normal	3	0:2:1:0	84.7 ± 12.9	68.7 ± 9.1	20.6 ± 2.2	2.5 ± 0.3	84.2 ± 21.9	3:0:0	0:3:0:0	5.7 ± 5.5(3)
	**Total**		**44**	**1:6:36:1**	**76.0 ± 10.5**	**68.9 ± 9.0**	**20.9 ± 2.3**	**4.2 ± 1.6**	**56.8 ± 24.9**	**25:15:4**	**11:24:5:4**	**4.8 ± 4.9(40)**
	**β**^**0**^**/HbE**	**Normal**	**27**	**0:11:13:3**	**72.2 ± 11.5**	**62.6 ± 7.7**	**18.7 ± 2.4**	**57.0 ± 9.6**^**5**^	**34.1 ± 11.4**	**10:15:2**	**9:9:7:2**	**4.5 ± 3.3(25)**
	β^+^/β^+^	α Thal^6^	1	0:0:1:0	73	74	24.5	3.3	50.9	0:1:0	0:0:1:0	15
	β^+^/β^+^	Triplication	1	0:0:1:0	76	76.2	27.3	3.4	65.2	1:0:0	0:0:1:0	NA
	β^+^/β^0^	α Thal^6^	6	0:2:4:0	78.8 ± 16.2	60.3 ± 6.1	19.4 ± 2.4	5.25 ± 1.8	73.1 ± 6.8	3:3:0	2:1:3:0	3.9 ± 1.4
	β^0^/β^0^	α Thal^6^	7	0:5:1:1	80.0 ± 11.8	67.1 ± 9.4	20.7 ± 2.4	4.1 ± 1.0	87.4 ± 21.7	2:5:0	1:2:4:0	6.3 ± 5.1(6)
	**Total**		**15**	**0:7:7:1**	**78.8 ± 12.5**	**65.5 ± 8.8**	**20.9 ± 3.1**	**4.4 ± 1.4**	**77.8 ± 18.4**	**6:9:0**	**3:3:9:0**	**5.9 ± 4.5(13)**
	β^0^/HPFH	Normal	7	0:4:2:1	78.9 ± 11.2	64.1 ± 5.0	20.2 ± 1.6	3.2 ± 1.5	96.8 ± 1.5	2:5:0	0:5:1:1	4.1 ± 1.5(5)
	β^0^/δβ	Normal	2	0:0:2:0	77.0 ± 1.4	75.4 ± 7.6	21.4 ± 0.6	0.6 ± 0.1	99.4 ± 0.1	2:0:0	0:2:0:0	NA
	β^0^orβ^+^/δβ	α Thal^6^	2	0:0:1:1	74.0 ± 1.4	63.4 ± 4.0	19.5 ± 2.0	3.2 ± 1.1	76.3 ± 0.1	0:2:0	0:2:0:0	2.3 ± 0.4
	**Total**		**11**	**0:4:5:2**	**77.6 ± 8.9**	**66.0 ± 6.6**	**20.3 ± 1.5**	**2.7 ± 1.6**	**93.5 ± 8.7**	**4:7:0**	**0:9:1:1**	**3.6 ± 1.5(7)**
**II**	β^+^/β^N^	Normal	1	0:0:1:0	89	64	21.9	5.5	2.1	0:1:0	0:0:1:0	10
	β^0^/β^N^	Normal	13	0:1:8:4	84.2 ± 9.6	57.3 ± 7.6	18.4 ± 2.1	5.0 ± 1.2	4.9 ± 3.6	1:12:0	0:5:8:0	7.3 ± 5.3(10)
	**Total**		**14**	**0:1:9:4**	**84.5 ± 9.3**	**57.8 ± 7.5**	**18.7 ± 2.2**	**5.0 ± 1.2**	**4.7 ± 3.6**	**1:13:0**	**0:5:9:0**	**7.5 ± 5.1(11)**
	β^0^/β^N^	Triplication	**5**	0:0:5:0	90.2 ± 14.2	60.0 ± 5.1	17.9 ± 1.2	6.0 ± 0.7	0.5 ± 1.0	0:4:1	0:0:5:0	12.3 ± 3.5(4)
	β^dominant^/β^N^	Normal	**1**	0:1:0:0	99	65.3	18.8	5.19	10.4	0:1:0	0:1:0:0	16

**I**: Type I TI (97,82.9%), **II**: Type II TI (20,17.1%). ^1^number of patients whose XmnI site were +/+:+/-:-/-:NA(NA, not available); ^2^number of patients in transfusion (T, transfusion; NT, not transfusion; NA, not available). ^3^number of patients with splenectomy (S, splenectomy; NS, hepatosplenomegaly but not splenectomy; NH, not hepatosplenomegaly; NA, not available). These three groups of data were described based on the patient's numbers, which were recorded by genotyping of Xmn1 polymorphism (Xmn1) or by positive scoring of recruited patients in clinical data collection (transfusion and splenectomy). The total number of each for these three groups of data should be equal to the number of patients in third colomn of this table. ^4 ^Mean ± SD of age at diagnosis (years); numbers in brackets indicate the number of patients. ^5 ^Including HbA_2 _and HbE. ^6^Including -α^3.7^,-α^4.2^,--^SEA ^and α^CS^

#### RNA analysis

Total cellular RNA was isolated from fresh peripheral blood using QIAamp RNA Blood Mini Kit (Qiagen, Germany). The cDNA synthesis was performed using the ExScript RT reagent Kit (TaKaRa Biotechnology, China). The expression levels of β-globin (target gene) and β-actin (control gene) were measured by SYBR Green-based relative quantitative RT-PCR assays described previously by Yipeng [28]. Five heterozygous subjects carrying the Cap+39(C→T) mutation and three heterozygous subjects carrying the Term CD+32(A→C) mutation were selected respectively, as two patient groups, while six normal subjects were used as the control group.

β-globin haplotype analysis: the β-globin haplotypes associated with the two novel mutations were analyzed with PCR amplification followed by restriction-enzyme digestion [28]. Seven classical polymorphic restriction enzyme sites selected for haplotype analysis were Hinc II-5'ε, Hind III-^G^γ, Hind III-^A^γ, HincII-ψβ, Hinc II-3'ψβ, Ava II-β and Bam HI-3'β. Each individual was scored for the presence (+) or absence (-) of each of the seven RFLP sites.

### Statistical methods

Statistical analysis was performed using SPSS software (Version 13.0, SPSS inc, USA). The difference of the relative mean mRNA concentration between mutation carriers and normal individuals were analyzed by the independent samples t-test. A *p*-value < 0.05 was considered as statistically significant.

## Results

### Molecular basis of 117 TI patients in southern China

In this study, 117 Chinese individuals between 2 and 60 years of age were enrolled to characterize the molecular basis of β-thalassemia intermedia in southern China. Their clinical, hematological and molecular data are summarized in table 2. According to their genotype of β-globin, we divided them into two major types: Type I β-thalassemia homozygotes or compound heterozygotes for β-thalassemia and other β-globin defects (Hb E, HPFH or δβ-thalassemia) (n = 97) who inherited two deficient β-globin alleles and Type II β-thalassemia heterozygotes (n = 20) who had only a single β-thalassemia allele. Statistical comparison showed that their steady-state Hb level of Type II (86.7 ± 10.8 g/L) was significantly higher than that of Type I (75.7 ± 11.4 g/L) (Mann-Whitney test, *p *< 0.05). In contrast, their HbF level of Type I (57.9 ± 27.2%) was much higher than that of Type II (4.0 ± 3.8%, *p *= 0.000). Significant differences in hematological parameters such as MCV (66.3 ± 8.7 fL in Type I vs. 58.7 ± 6.9 fL in Type II, *p *= 0.004) and MCH (20.2 ± 2.5 pg in Type I vs. 18.5 ± 2.0 pg in Type II, *p *= 0.000) were also observed. In total, we detected 18 β-thalassemia alterations including two novel ones which were termed as Term CD +32(A→C) and Cap+39(C→T), β-globin variant HbE, the two deletional mutations which can lead to SEA-HPFH or (δβ)^0^-thalassemia Chinese ^G^γ^+^(^A^γδβ)^0 ^(table 3).

**Table 3:** β globin mutations identified in 117 patients with TI phenotype.

Mutation	Type of thal	Location	No. of Chr.		Total (%)
			Type I	Type II	
-28 (A→G)	β^+^	5'UTR	42	1	43(20.1%)
CD 41-42(-CTTT)	β^0^	Exon	39	3	42(19.6%)
CD 17 (A→T)	β^0^	Exon	29	5	34(15.9%)
CD 26 (G→A)	Hb E	Exon	27	0	27(12.6%)
IVS-2-654 (C→T)	β^0^	Intron	12	8	20(9.3%)
IVS-2-5 (G→C)	β^+^	Intron	11	0	11(5.1%)
CD 71/72 (+A)	β^0^	Exon	6	2	8(3.7%)
SEA-HPFH	HPFH		7	0	7(3.3%)
IVS-1-1 (G→T)	β^0^	Intron	5	0	5(2. 3%)
-29(A→G)	β^+^	5'UTR	5	0	5(2.3%)
Chinese ^G^γ^+^(^A^γδβ)^0^	δβ thal		4	0	4(1.9%)
CD 43 (G→T)	β^0^	Exon	2	0	2(0.9%)
-73(A→T)	β^+^	5'UTR	1	0	1(0.5%)
Term CD +32(A→C)^1^	β^+^	3'UTR	1	0	1(0.5%)
Cap+39 (C→T)^2^	β^++^	5'UTR	1	0	1(0.5%)
CD 15/16 (+G)	β^0^	Exon	1	0	1(0.5%)
CD 27/28(+C)	β^0^	Exon	1	0	1(0.5%)
CD 53(-T)	β^dominant^	Exon	0	1	1(0.5%)
Total number of chromosomes			194	20	214(100%)

^1,2 ^Novel mutations found in this study.

Type I β-thalassemia homozygotes or compound heterozygotes for β-thalassemia and other β-globin defects were detected in 97 TI patients (82.9%) as follows: (i) Fifty-nine (50.4%) had homozygous or compound heterozygous β-thalassemia alleles with co-existing normal (44 cases, 37.6%) or mutant (15 cases, 12.8%) α-globin genes. Four types of the α-thalassemia alterations were identified in our TI patients, including the -α^3.7^, -α^4.2^, --^SEA ^alleles or α^CS^α. We observed two TI patients with particular combinations of α-globin defects and common β-globin genotypes. One was caused by co-existence of Hb H disease (--^SEA^/-α^4.2^) and β-thalassemia compound heterozygosity CD17 (A→T)/IVS-2-654 (C→T), while the other was caused by αα/ααα^anti-3.7 ^and β-thalassemia homozygosity of the -28 (A→G) mutations. The first case was a male child with Hb 66 g/L and a splenectomy, when he was diagnosed at the age of 3 years, he had been received a transfusion only one time. His blood indices showed RBC 4.13×10^12^/L, MCV51.7fL, MCH16.0 pg, HbA_2 _4.0%, and HbF38.1%. The second case was a 12-year-old boy who had never been transfused. Pallor, hepatosplenomegaly and slight facial alterations were noted. He had presence of blood indices with microcytic-hypochromic anemia: Hb76 g/L, MCV76.2fL, MCH27.3 pg, HbA_2 _3.4% and HbF65.2%. (ii) Twenty-seven (23.1%) were HbE/β^0 ^compound heterozygotes. No HbE/β^+ ^heterozygote or co-incident α-globin mutations were found in this group. (iii) Eleven (9.4%) carried SEA-HPFH (7 cases) or Chinese ^G^γ^+^(^A^γδβ)^0 ^(4 cases) deletions in addition to having one β-thalassemia mutation. Among them, two were IVS-2-654 (C→T)/Chinese ^G^γ^+^(^A^γδβ)^0 ^and -28 (A→G)/Chinese ^G^γ^+^(^A^γδβ)^0^, respectively, both plus one --^SEA ^deletion, which were first reported by Li in our lab [22].

Type II β-thalassemia heterozygosity was detected in 20 TI patients (17.1%) as follows: (i) Fourteen (12.0%) carried a single β-thalassemia allele and normal α-globin genes. (ii) Five (4.2%) had a single β-thalassemia allele and co-inherited ααα^anti-3.7 ^triplication. No ααα^anti-4.2 ^triplication was detected. (iii) One patient (0.9%), in a Miao family, was a heterozygote for a frameshift β-thalassemia mutation at CD 53 (-T). This mutation is considered to be a dominant form and was first reported by Yi in our lab in 2008[28].

### Analysis of genotypic modifiers in 3 β^0^/β^0 ^and 10 β^0^/β^N ^or β^+^/β^N ^TI cases

Usually, thalassemia patients with β^0^/β^0 ^genotype and normal α-globin gene had the TM phenotypes, but we detected 3 β^0^/β^0 ^patients with TI phenotypes whose Hb levels are 74-99 g/L (table 4). We examined the existence of known genetic modifiers, which related to increasing HbF levels in these three cases. The clinical, hematological and molecular results observed from the group of patients are listed in table 4.

**Table 4:** Hematological data and existence of genetic modifiers identified among 3 β^0^/β^0 ^TI patients

Studied items	Data of patients		
	1	2	3
Age/Age at diagnosis (years)	7/2	5/3	19/12
Age of 1^st ^Transfusion (years)/frequency	4/1 time every 7 months	4/1 time every 6 months	17/only 1 time
Hepatosplenomegaly^1^	mild	2/2(cm)	0.5/8(cm)
RBC(10^12^/L)	3.64	3.51	5.32
Hb (g/L)	74	81	99
MCV (fL)	70	77	59
MCH (pg)	20.3	23.0	18.6
MCHC (g/L)	289	298	313
HbF (%)	96.4	59.0	97.3
HbA_2 _(%)	2.1	2.7	2.7
β-globin genotype^1^	CD 17 (A→T)/IVS-2-654 (C→T)	IVS-1-1 (G→T)/CD 41-42 (-CTTT)	CD 17(A→T)/CD17 (A→T)
*Xmn I*	+/-	+/-	-/-
β-540	(AT)7(T)5/(AT)8(T)5	(AT)7(T)7/(AT)8(T)5	(AT)7(T)5/(AT)7(T)5
5' HS2	(AT)9(N)12(AT)10/(AT)8(N)12(AT)10	(AT)9(N)12(AT)10/(AT)8(N)12(AT)10	(AT)9(N)12(AT)11/(AT)8(N)12(AT)11

^1 ^numbers before or after the slash indicate the enlarged liver or spleen in size (cm: centimeter) when first diagnosis. The α-globin genotype, the sequence analysis of Aγ-(-500~+223) and Gγ-(-618~+39) globin genes are all normal in the three samples. The genotypes of both 3'HS1(+179) polymorphic site and rs11886868 SNP site of *BCL11A *gene are all C/C in the three samples.

Fourteen patients who displayed the relative mild TI phenotypes with steady Hb levels of 68-95 g/L were identified to be heterozygotes with one known common mutation for β-thalassemia and normal α-globin gene. Analysis of other known genetic modifiers that mainly focused on several factors of aggravate α/β imbalance were done in ten patients (blood samples from other four patients were not available). The overall findings are summarized in table 5.

**Table 5:** Htological data and existence of genetic modifiers identified among 10 β^+^/β^N ^or β^0^/β^N ^TI patients

Studied items	Data of patients									
	1	2	3	4	5	6	7	8	9	10
Age/Age at diagnosis (years)	26/10	17/5	7/7	5/4	26/8	7/7	5/5	12/12	6/3	38/20
Transfusion	No	No	No	No	Occasionally	No	No	No	No	No
Hepatosplenomegaly^1^	No	No	No	No	0.5/6(cm)	No	mild	No	No	No
RBC(10^12^/L)	4.05	5.83	5.16	5.52	3.33	5.37	5.75	5.77	5.03	4.57
Hb (g/L)	89	88	91	88	68	94	95	84	82	92
MCV (fL)	64	49	50	51	68	53	54	60	54	57
MCH (pg)	21.9	15.0	17.6	15.9	20.4	17.5	16.5	19.1	16.3	20.1
MCHC (g/L)	342	307	350	314	298	328	305	319	302	355
HbF (%)	2.1	6.2	2.6	2.2	8.1	1.3	4.3	0	1.5	2.5
HbA_2 _(%)	5.5	4.3	5.2	5.8	2.6	5.6	5.1	5.8	5.5	5.2
β-globin genotype	-28 (A→G)/N	CD 17 (A→T)/N	CD 17 (A→T)/N	CD 17 (A→T)/N	IVS-2-654 (C→T)/N	IVS-2-654 (C→T)/N	CD41-42 (-CTTT)/N	IVS-2-654 (C→T)/N	CD71-72 (+A)/N	CD71-72 (+A)/N
*Xmn I*	-/-	+/-	-/-	-/-	-/-	-/-	-/-	-/-	-/-	-/-
*GATA-1*	Normal	Normal	Normal	Normal	Normal	Normal	ND	ND	ND	ND
*HRI*^2^	(T/T) (T/T)	(T/C) (T/T)	(T/C) (T/C)	(T/C) (T/T)	(T/C) (T/T)	(C/C) (C/C)	ND	ND	ND	ND

^1 ^numbers before or after the slash indicate the enlarged liver or spleen in size (cm: centimeter) when first diagnosis.^2 ^Genotypes of two SNP sites including rs2639 and rs2640 detected in the HRI cDNA. ND: not determined,because of no mRNA available for these samples. The α-globin genotype, analysis of core regions of both 5'HS2 and 5'HS3, and *AHSP *gene are all normal in the tested ten samples. Blood samples from other four out of fourteen TI patients with heterozygous genotypes were not available, thus the later three targets in the table can not be conducted for these four patients.

### Two novel mutations of the β-globin gene

**Figure 1:**
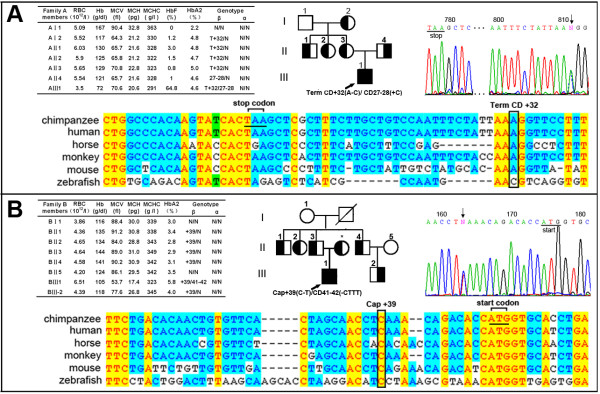
**Identification of two novel mutations showing the (A)Term CD+32(A-C) and the (B) Cap+39(C-T). **The hematological data and α/β globin genotype of two family members are listed in tables. The probands are labeled by the arrow in the family pedigrees. DNA sequences of sense strand are also shown, downward arrows indicating the mutation nucleotides (overlapping peaks are indicated by N). The alignment of β-globin sequences are shown on the bottom. (EMBL accession No.: chimpanzee ENSPTRT00000006177, human ENST00000335295, horse ENSECAT00000010442, monkey ENSMMUT00000006876, mouse ENSMUST00000098192, and zebrafish ENSDART00000101713). *(Blood samples not avaiable). T+32: Term CD+32(A-C), 27-28:CD 27-28(+C), +39:Cap+39(C-T), and 41-42: CD 41-42 (-CTTT).

Two novel mutations, Term CD+32(A→C) (GenBank No: FJ876836) and Cap+39(C→T) (GenBank No: FJ876835), were identified in this study (Figure 1).

Family A: Term CD+32(A→C). The proband (AIII1) displayed the TI phenotypes with Hb 72 g/L, HbA_2 _4.6% and HbF 64.8%. His father was found to have a CD 27-28 (+C) mutation, whereas his mother was found to have a novel mutation of an A→C substitution at nucleotide position +32 from the stop codon TAA in the 3' UTR region of the human β-globin gene (Figure 1A). The proband was a compound heterozygote of Term CD+32(A→C)/CD 27-28(+C) with *XmnI *(+/+) homozygosity. The haplotypes associated with Term CD+32(A→C) and CD 27-28(+C) are "--+++-+"and "--++++-", respectively.

Family B: Cap+39(C→T). The proband (BIII1) showed a mild TI phenotypes with Hb 105 g/L, MCV 53.7 fL, MCH 17.4 pg, HbA_2 _5.8%. His father had a novel mutation of a C→T substitution at nucleotide position +39 from the cap site in the 5' UTR region of the human β-globin gene. This mutation was likely inherited from his dead grandfather. His mother did not provide her blood sample, but she was an obligate carrier with CD 41-42(-CTTT) mutation since her son (BIII1) was a compound heterozygote of Cap+39(C→T)/CD 41-42(-CTTT) with *Xmn I *(-/-) homozygosity. Interestingly, his father (BII3) and four other family members (BII1, BII2, BII4, and BIII2) were all heterozygotes of Cap+39 (C→T) mutations. Since they do not have any evident hematologic phenotype, we regard this mutation as β^++ ^(silent β-thalassemia).

Sequence alignment showed that Term CD+32(A) and Cap+39(C) were conserved sites during evolution (Figure.1). In addition, we randomly choosed 156 cases normal individuals from Guangxi as control samples and excluded the possibility of that the two new mutations were polymorphic sites. These control samples are 13-19 years old with Hb (130-152 g/L), MCV (83-100 fL), MCH (28-33.8 pg), HbA (96.54-97.94%) and HbA_2 _(2.06-3.46%).

Real-time PCR was used to analyze the mRNA level. Two standard curves were generated: *y *= -3.672*x *+ 19.787 (*R*^*2 *^= 0.999) for β-globin mRNA and *y *= -3.466*x*+24.000 (*R*^*2 *^= 0.999) for β-actin mRNA. The mean relative mRNA concentrations were 0.835 ± 0.048 (*n *= 3) for the Term CD+32(A→C) group, 1.093 ± 0.118(*n *= 5) for the Cap+39(C→T) group, and 1.016 ± 0.098 (*n *= 6) for the normal control group after a normalization procedure using linear regression equations. Statistical analysis showed that there was a significant difference (*p *= 0.021) of mean relative β-globin mRNA concentration between the Term CD+32(A→C) group and the control group. The decreased level of β-globin mRNA in the patient group compared with that of the normal control group was calculated to be 17.8%. In contrast, no significant difference (*p *= 0.270) was found between the Cap+39(C→T) group and the control group.

## Discussion

Provision of appropriate treatment and management for TI patients is still a challenge because its characteristic features, phenotypic diversity, and genotypic heterogeneity, the clinical severity of thalassemia intermedia cases are very difficult to predict from their genotypic data. The molecular mechanisms underlying different populations in Europe [18,20], Israel [19], India [17] and Iran [16] had been reported. However, the molecular basis of Chinese TI patients was still uncertain, which brought about much trouble for Chinese doctors in offering genetic counselling, treatment and management. The present phenotype-genotype correlation data observed from 117 Chinese TI patients could provide solid basis for improving the understanding this problem (table 2). On the basis of the clinical pictures and statistical results of hematological parameters, type I patients were more clinically severe than type II patients. This is a patient-based study in which the in-patient samples were recruited during the period of about 2 years from Guangxi and Guangdong Provinces, where the thalassemia are most endemic in southern China. We believe that the molecular basis of TI obtained from this study should be generalizable to the Chinese TI patients of the various regions in southern China because the differences between regions in southern China for the ß-thalassemia mutation spectrum in the southern Chinese are not statistically significant according to previously observations [1-3,5]. However, there were some milder forms of TI patient's samples who were out-patient we might have missed and most of heterozygotes (10/14) whose negative results could not be explained were detected in Guangxi, two limitations of our study should be noted: (1) Maybe there was a broad spectrum of genetic defects in the Chinese TI patients, such as no the HbE/β^+ ^TI patients could be identified in our cohort. (2) The finding of heterozygotes with TI phenotype might not provide representitive profile in the Chinese population.

According to our molecular analysis, three main characteristic features of Chinese TI patients were found. First, the homozygous or compound heterozygous forms with minor β-thalassemia mutations (the β^+^-thalassemia alleles) were a common pathogenetic mechanism of disease in Chinese TI patients, in which 49 out of 59 patients was found to having β-globin genotypes of β^+^/β^+ ^(11 cases) and β^+^/β^0 ^(38 cases), among them 41 with co-existing normal α-globin genes and 8 with mutant α-globin allele (table 2). In other words, the β^+^-thalassemia mutation is a major contribution of genes to the Chinese TI patients. In addition to this, additional an α-thalassemia defects may reduce the severity of homozygous β-thalassemia. We observed 14 such cases (23.7%, 14/59) with co-inheritance of β-thalassemia (β^+^/β^+ ^or β^+^/β^0^) and α-thalassemia defects in the patient group. Second, the affect of an *XmnI *polymorphism in increasing HbF levels was not as important as it was in other populations. The ratio of *XmnI *[(+/+), (+/-), (-/-) and (NA, not available)] pattern was detected to be [1:30:75:11] in 117 cases, while the same ratio in 52 Iranian TI patients was [20:20:10:2] [16]. In India, among 73 TI patients, 20 of them (27.4%) were confirmed to be homozygous for the *XmnI *polymorphism [17]. Third, the molecular mechanism of more than half of the thalassemia heterozygote cases (14/20) was unexplained. Twenty individuals carried only one β-thalassemia allele: one (5%) was dominantly inherited; five (25%) had co-inherited triplicated α-globin genes; and 14 (70%) had normal α-globin genes. In contrast, the α-globin gene triplication was determined to be the predominant factor of Indian heterozygous thalassemia patients (14/23 cases) [17]. In Ho's research, among 22 heterozygous β-thalassemia intermedia cases, 10 patients had α triplication [20]. Furthermore, no HbE/β^+ ^heterozygote was observed in our cases while 9/45 TI patients were HbE/β^+ ^heterozygotes in Thailand [29], suggesting that HbE/β^+ ^heterozygosity perhaps displayed a more milder form of TI in Chinese people, which would help doctors in providing appropriate genetic counselling. HbE/β-thalassemia results in a varied clinical expression ranging from severe transfusion dependence to a complete lack of symptoms [29,30]. In this study, there are 27 cases HbE/β^0 ^TI patients whose clinical phenotypes including Hb levels, transfusion, splenectomy, age at diagnosis, and so on, varies greatly (table 2). It is very difficult to predict the clinical phenotype of the HbE/β-thalassemia although it was reported that a system for classifying disease severity of HbE/β-thalassemia had been constructed [29,31]. The possible genetic mechanisms for phenotypic diversity of HbE/βthalassemia have been analyzed in some literatures [29,30,32]. We also found two patients that were compounds of β-thalassemia, δβ-thalassemia and α-thalassemia genes. This was rarely reported in other ethnicities.

Three β^0^/β^0 ^TI patients with normal α-globin gene remained unexplained despite extensive effort to examine the existence of known genetic modifiers linked to increase HbF levels (table 4). The non-deletional forms of HPFH were excluded in sequencing the promoter region of the ^G^γ- and ^A^γ-globin genes. Two cases were +/- of *XmnI*, which partially, but not completely explains the high level of HbF. The 3'HS1 (+179 C→T) and rs11886868 (T→C) variations were shown to correlate with increased HbF in European TI patients [8,11]. The three patients all had C/C genotypes of both at +179 of 3'HS1 and rs11886868. However, we could not consider that C/C genotypes of rs11886868 contributed to the high HbF level based on two facts. One is that the frequency of C/C genotypes was 0.867 in Chinese people from the HapMap data http://www.hapmap.org/. The second is that an additional 115 samples were analyzed, including 30 TI patients (25 β^0^/β^+^, 5 β^+^/β^+^) and 85 normal individuals, in which all were found to be the C/C genotype (data not shown). The (AT)_8_N_12_(AT)_11 _motif in the 5'HS2 site and the (AT)_9_(T)_5 _motif in the -540 region had been previously found to be associated with elevated HbF [9,10], but we did not find them in the 3 cases.

We found 14 patients with heterozygous for β-thalassemia among our 117 TI patient cohort, in whom the β-globin gene was found to be structurally intact by sequence analysis and the second α-globin gene triplication was excluded. They mostly manifested a relatively mild form of TI because of the Hb levels being 68-95 g/L, the average age at diagnosis being 7.5 ± 5.1 (years), no blood transfusion or occasionally received (1/14) and mild hepatosplenomegaly (5/14). As for the underlying defective targets, we focused on primary genetic modifiers that could modulate the imbalance of α/β for investigation in ten TI patients (table 5). We evaluated the effects of some potential modifiers involving one linked to the β-globin gene cluster, the LCR resides in the 5'HS2 and 5'HS3 regions [33], and three ones not to be linked to the β-globin cluster, AHSP [14], GATA-1[12] and HRI. The later is a kinase that can be inactivated by hemin, which is very recently reported to modify the phenotypic severity of murine models of erythropoietic protoporphyria and β-thalassemia [15]. Sequence analysis of the core regions of 5'HS2 and 5'HS3 showed wild type sequences, thus excluded the possibility that mutations in these control regions reducing the expression of β-globin gene. No mutations in the cDNA sequences of both AHSP and GATA-1 have been found. In the case of HRI, the polymorphism of rs2639 and rs2640 were detected. The rs2639 (A→G) is a synonymous mutation and the rs2640 (A→G) is a missense mutation resulting in a K558R substitution. However, the affect on the function of this substitution is unknown since Lys (K) and Arg (R) are similar in amino acid properties. These results indicate the existence of causative genetic determinants have not been defined molecularly, in agreement with the similar conclusion from Rosatelli's experiment [34] although there were a few differences between the candidate targets for these two studies.

In this study, we reported two novel mutations in the UTR regions of the human β-globin gene (Figure.1). The Term CD+32(A→C) mutation slightly reduced β-globin mRNA levels to 17.8% less than the normal level. The reduced β-globin mRNA levels were not detected in the individuals with Cap+39(C→T) mutation. The detailed mechanisms of two novel mutations are not clear at present. Functional experiments, such as analysis of mRNA stability and trafficking in erythrocytic cells should be performed in the future.

## Conclusions

Chinese TI patients showed a high degree of heterogeneity in both phenotypic and genotypic aspects. Some cases could not be explained according to the present data. A more detailed analysis of genetic modifiers modulating the imbalance of α/β will provide more insight into the recognition of thalassemia intermedia. Moreover, other potential genetic determinants which have been demonstrated in cancers and single gene disorders including α- and β-thalassemia, such as a gain-of-function regulatory SNP in a nongenic region [35], loss of heterozygosity in the β-globin gene [36], segmental duplications involving the α-globin gene cluster [37], uniparental disomy event[38], DNA methylation and chromatin alterations, should also be considered.

## Competing interests

The authors declare that they have no competing interests.

## Authors' contributions

WC, XZ and XS contributed equally to the work. XX conceived and designed the study and manuscript revision. WC designed and performed the whole experiments, analyzed the data and wrote the manuscript. XZ collected and analyzed clinical and hematological data of the patients. XS participated in analyzing the results and writing the manuscript. RC and TZ assisted in blood sample collection and clinical data analysis. LL, MS and FX participated in partial molecular experiments. All authors read and approved the final manuscript.

## Pre-publication history

The pre-publication history for this paper can be accessed here:

http://www.biomedcentral.com/1471-2350/11/31/prepub
